# Modular and configurable optimal sequence alignment software: *Cola*

**DOI:** 10.1186/1751-0473-9-12

**Published:** 2014-06-09

**Authors:** Neda Zamani, Görel Sundström, Marc P Höppner, Manfred G Grabherr

**Affiliations:** 1 Science for Life Laboratory, Department of Medical Biochemistry and Microbiology, Uppsala University, Uppsala, Sweden

**Keywords:** Optimal sequence alignment, Algorithm, DNA, Cola

## Abstract

**Background:**

The fundamental challenge in optimally aligning homologous sequences is to define a scoring scheme that best reflects the underlying biological processes. Maximising the overall number of matches in the alignment does not always reflect the patterns by which nucleotides mutate. Efficiently implemented algorithms that can be parameterised to accommodate more complex non-linear scoring schemes are thus desirable.

**Results:**

We present *Cola*, alignment software that implements different optimal alignment algorithms, also allowing for scoring contiguous matches of nucleotides in a nonlinear manner. The latter places more emphasis on short, highly conserved motifs, and less on the surrounding nucleotides, which can be more diverged. To illustrate the differences, we report results from aligning 14,100 sequences from 3' untranslated regions of human genes to 25 of their mammalian counterparts, where we found that a nonlinear scoring scheme is more consistent than a linear scheme in detecting short, conserved motifs.

**Conclusions:**

*Cola* is freely available under LPGL from https://github.com/nedaz/cola.

## Findings

The fundamental question for optimal alignment of genomic sequences is how to define "optimality", with particular regard to biological relevance. Mathematically, optimality is determined by the underlying scoring function, and different linear and limited nonlinear schemes have been proposed [1-7]. To date, the Smith-Waterman gap affine (SWGA) method [3], a modification of the Smith-Waterman (SW) method [7] that applies additional penalties to alignment gaps regardless of the gap size itself, has remained the method of choice, and is utilised by common alignment tools [8]. Here, we extend this method (SWGA+) by allowing for any arbitrary nonlinear match scoring function, enabling us to give higher weights to consecutive matching nucleotides, rather than optimising the total number of matches. The software we have developed, *Cola* (Contiguous optimal local aligner), is a C++ implementation of this algorithm, which, by applying a linear match scoring function, degenerates into the SWGA and SW schemes. Notable features include a constant factor difference between generalised and linear scoring, both in runtime and memory consumption. Also, banded alignments, i.e. limiting the search space to a band around the diagonal, allows for scaling linearly in time with sequence length. We also utilise a variation of an algorithm that significantly improves the space complexity from O(N^2^) to O(2 N). This is based on the check-pointing method introduced by Powell et al. [9] as an extension of the divide-and-conquer method introduced by Hirschberg [10].

*Cola* provides an Application Programmer’s Interface (API) for ease of integration into larger software packages, and is e.g. included in the recent versions of the whole genome synteny aligner *Satsuma*[11], and the universal genome coordinate mapper *Kraken* (Zamani et al., submitted). Depending on parameterisation, *Cola* is bit-compatible with the SW and SWGA methods.

## Methods

**Figure 1:**
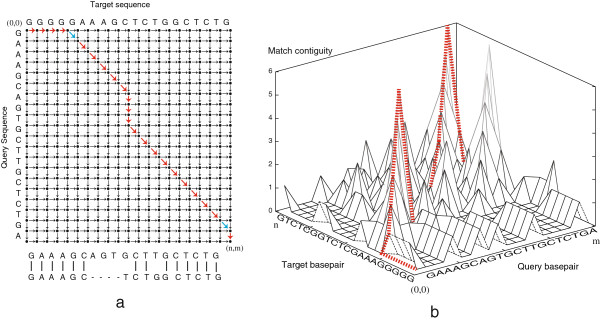
**2D and 3D edit graphs. (a)** Edit-graph for the alignment between the target and query sequences shown on the top and left hand side of the graph. The optimal path chosen by SWGA+ is shown with red arrows and the corresponding alignment is shown below the graph. Note that the local alignment starts and ends with the blue arrows. **(b)** The corresponding 3D edit-graph for the alignment shown in part **(a)**. For better visibility the starting point, (0,0), in this graph is shown on the bottom-left and end point (n,m) is on the top-right. Each cell contains n number of nodes, which are shown on the third dimension of the graph and correspond to the lengths of contiguity starting from 0 to di,j. The optimal path, which is similar to the path shown in part **(a)** is depicted in a dotted red line.

Both SW and SWGA maximise the overall number of matches, modulo the gap penalty, in a linear fashion, e.g. each nucleotide match or mismatch is scored separately, conceptually allowing for computing the alignment on a two-dimensional grid. Cola implements this strategy, but extends the dimensionality to accommodate non-linear scoring of consecutive matches so that confined sequence motif matches are preferred over dispersed matches within their diverged flanks. Since this algorithm adds considerable computational complexity, i.e. the alignment can no longer be performed in two dimensions, *Cola* implements a three-dimensional graph structure analogous to the 'edit-graph' used by SW and SWGA. The difference is exemplified in Figure 1: in two dimensions, every node in a graph can be reached from a preceding neighbour by means of a horizontal, vertical, or diagonal edge (Figure 1a). Assuming that the query sequence is represented horizontally and the target sequence vertically, the horizontal, vertical, and diagonal edges entering a node, in turn, correspond to an insertion or deletion (indel) in the query, indel in the target, and a match or mismatch between the query and target bases at the node coordinates. In three dimensions, SWGA keeps nodes for vertical and horizontal moves separately at each cell position (i,j), storing the number of consecutive matches in the third dimension (Figure 1b), so that scores are computed in any arbitrary manner and can depend on the match depth, allowing for favoring fewer consecutive matches over more total matches.

Cola implements its search method using a dynamic programming algorithm, similar to SWGA, but with the added challenge of tracking match contiguity. The recurrence given in the equation below keeps the runtime almost as that of SWGA, affecting it only by a linear factor (See section on performance for more detail).

(1) $$S_{i,j}=\max_{n=0:d_{i,j}}\left\{\begin{array}{l} S_{i,j,n}\\ {\vec{S}}_{i,j,0}\\ {\overset{↓}{S}}_{i,j,0}\\ 0 \end{array}\right)$$

*S*_*i*,*j*_ represents the score pertaining to the optimal local alignment that extends to the *i*^*th*^ element of the target and *j*^*th*^ element of the query. This score is obtained by finding the optimum among all possibilities of reaching the node (*i*, *j*) from its neighbours, which are shown as four elements in Eq. 1. The first element *S*_*i*,*j*,*n*_ represents the score for reaching (*i*, *j*) with *n* number of contiguous matches, where *n* is enumerated from 0 to *d*_*i*,*j*_, the maximum number of contiguous matches possible, or in other words, the depth of the edit-graph at (*i*, *j*). The recurrence for computing *S*_*i*,*j*,*n*_ is shown below (Eq. 2), where *Δf*(*n*) = *f*(*n*) − *f*(*n* − 1) is the score difference for *n* and *n*-1 matches, which is added to *S*_*i* − 1,*j* − 1,*n* − 1_ to obtain *S*_*i*,*j*,*n*_, and *f*(*n*) can be any arbitrary function. If *n* = 0, i.e. the target base *i* and query base *j* do not match, a fixed mismatch penalty *ρ*_*m*_ is applied.

(2) $$S_{i,j,n}=\left\{\begin{array}{cc} S_{i-1,j-1,n-1}+\mathrm{\Delta f}\left(n\right) & n>0\\ S_{i-1,j-1}-\rho_{m} & n=0 \end{array}\right)$$

The next two elements in Eq. 1 are ${\vec{S}}_{i,j,0}$and ${\overset{↓}{S}}_{i,j,0,}$ which represent the horizontal and vertical moves, i.e., indels kept separately for enabling gap-affine scoring. The recurrence relationships below demonstrate how these values are calculated, where *ρ*_*o*_ is the cost of opening a gap and *ρ*_*e*_ is the cost for extending a gap.

(3) $${\vec{S}}_{i,j,0}=\max\left\{\begin{array}{c} {\vec{S}}_{i-1,j}-\rho_{e}\\ S_{i,j-1}-\left(\rho_{o}+\rho_{e}\right) \end{array}\right)$$

(4) $${\overset{↓}{S}}_{i,j,0}=\max\left\{\begin{array}{c} {\overset{↓}{S}}_{i,j-1}-\rho_{e}\\ S_{i-1,j}-\left(\rho_{o}+\rho_{e}\right) \end{array}\right)$$

The last element in Eq. 1, i.e., 0 is required for finding the local alignment as opposed the global. It translates into starting the alignment from any point that maximises the overall alignment score. Removing this element from the recurrence turns the algorithm into a global aligner rather than a local one.

To conceptualise the effect of the aforementioned recurrences, we can think of extending the SWGA edit-graph structure by appending nodes that correspond to the cost of getting from cell (i,j) to cell (i', j') with n contiguous matches. This translates to adding one node for each value of n at every cell (i,j) in the graph. Conceptually, the underlying edit-graph becomes three-dimensional, with the third dimension containing the nodes that track the path and cost of contiguous matching. For memory efficiency, Cola dynamically allocates the third dimension only in an on-demand fashion (see Figure 1b), and limits the depth to 1 in case a linear function is applied (e.g. to match the SW and SWGA schemes). The depth at each cell (i,j) demonstrates the various lengths of contiguity of matches for which the best path can be obtained from cell (0,0) to cell (i,j). Knowing the length of contiguity of matches at every given node allows for applying a scoring mechanism based on an arbitrary function of the matches. *Cola* implements a cubic function _f (n) = n3 by default_, as manually examined examples suggest good balance between a too steeply rising function (e.g. exponential function) and a lower degree polynomial (e.g. quadratic function) in combination with the penalties *ρ*_*m*_= 8, *ρ*_*o*_= 200, and *ρ*_*e*_= 20, which we determined empirically (2 consecutive matches equal 1 mismatch penalty, 3 consecutive matches score higher than a single gap extension penalty, etc.).

## Performance and accuracy

**Figure 2:**
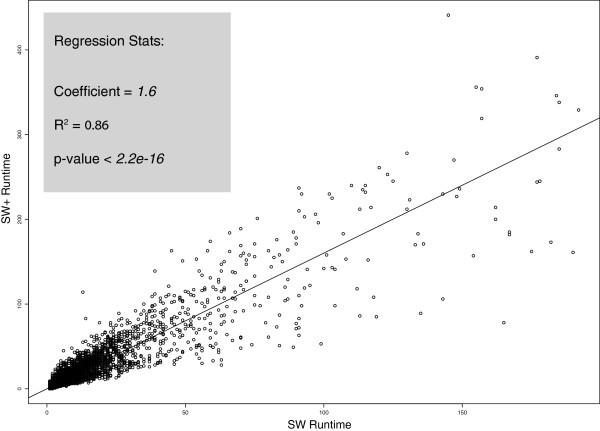
**The regression of SW+ runtime values against SW runtime for regions in the human and dog genome that were identified by BLAST to have a significant alignment score.** For this dataset, which is representative of others with similarly significant alignments, the SW+ runtime is by a factor of 1.6 slower than SW.

The time and space complexities of SW+ in comparison with SW are affected by a constant factor. The algorithm complexity for SW+ is in the order of O(kNM) as opposed to O(NM) for SW, where N and M represent the size of the target and query sequences and k is the average length of contiguity over the entire search space. The exact value of the constant factor k depends on the data, but is in practice below 2. To obtain an empirical measure of this coefficient, we show a graph of runtime of SW+ versus SW for aligning all regions in the human and dog [12] genomes that are identified by LASTZ chains [13] to be orthologous. From this dataset, consisting of about 500,000 alignments, we estimate k from a regression to be 1.6, as shown in Figure 2, along with significance statistics.

While the true accuracy of a pairwise alignment is difficult to assess, we define several measures to highlight the difference as a result of linear or non-linear scoring: (i) the number of positions in which all or most genomes agree with the reference, representing events that could be less likely to occur by random chance; and (ii) the number of elements consisting of n consecutive nucleotides matching all or most genomes. As a test set, we choose 14,100 3' Un-translated Regions (3'UTRs) of human coding genes that are between 100 and 1000 in size, with 4.9 million nucleotides in total sequence, and their orthologous sequences in at least 25 mammalian genomes [13]. 3'UTRs are known to contain short (6 nucleotides), and in some cases highly conserved recognition motifs required for post-translational processing of RNAs, including the poly-adenylation signal AATAAA.

**Figure 3:**
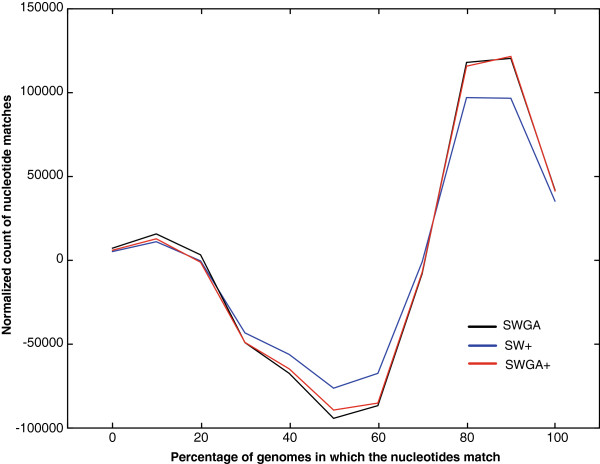
**Normalised counts of nucleotide matches over the percentage of genomes in which the nucleotides match.** Shown are the count distributions form SWGA (black), SW+ (blue), and SWGA+ (red), minus the counts for SW. All three scoring schemes exhibit a pronounced shift into the higher percentage bins compared to SW, with aligning fewer nucleotides that match fewer genomes (most notably the drop at the 50% mark), and more nucleotides that match in 80-100% of the genomes.

In comparing the different scoring schemes (restricted to the regions in which all methods report alignments), SW finds the largest number of matching nucleotides in all pairwise mammal-to-human alignments (see Table 1). The SW method yields fewer positions in which a higher fraction of genomes agree compared to SWGA, SW+ and SWGA+. Figure 3 shows the nucleotide counts over the percentage of matching genomes for SWGA, SW+, and SWGA+, after subtracting the SW counts. All three methods show a clear shift towards the higher end, indicating that the SW scoring method is sub-optimal in terms of consistency across multiple alignments. The SWGA and SWGA+ methods perform almost equally well in terms of single nucleotides. However, when examining the number of elements of 6 consecutive nucleotides (the size of the poly-adenylation signal) or longer that are matched in 90% of the aligned genomes or more, both SW+ and SWGA+ find more instances, as well as more total sequence in such regions (Table 1). When computing the frequencies of all 6-mers in these regions, we find that all methods report the poly-adenylation signal AATAAA to be the motif with the highest occurrence rate, and that SWGA+ finds 33 more instances than SWGA (Table 1), which is to be expected due to its scoring preference to compact motifs.

**Table 1:** Comparison of SW, SWGA, SW + and SWGA+ with regards to alignment consistency

**Alignment method**	**Matched nucleotides**	**Number of motifs**	**Sequence in motifs**	**Poly-adenylation sites**
SW	142,965,865	50,022	660,695	2,104
SWGA	138,315,733	51,951	697,997	2,277
SW+	142,231,531	53,135	704,241	2,291
SWGA+	138,944,794	53,609	710,514	2,310

Shown are the number of matching nucleotides in pairwise alignments, the number of motifs that are six nucleotides or longer and present in at least 90*%* of aligned genomes, the total sequence in such motifs, and the counts of the known poly-adenylation signal AATAAA.

## Software availability and requirements

*Cola* is an object-oriented software written in C++, compiled using gcc, and runs on Linux operating systems. The software is modular and provides a collection of utilities for input data conversion. The source code is designed to be easily configurable allows for easy integration as the back-end into e.g. seed-finding methods, such as BLAST (Stephen F [14]).

The source code is freely available under LPGL. For code usage guide, command line parameters, and output file formats, see website at https://github.com/nedaz/cola.

## Competing interests

The authors have declared that no competing interests exist.

## Authors’ contributions

NZ and MGG designed and developed the software and designed and performed the analyses. MPH, GS, and MGG provided the biological interpretation of the results. All authors wrote the manuscript. All authors read and approved the final manuscript.
